# How do terrestrial wildlife communities respond to small‐scale *Acacia* plantations embedded in harvested tropical forest?

**DOI:** 10.1002/ece3.9337

**Published:** 2022-09-20

**Authors:** Seth T. Wong, Roshan Guharajan, Azrie Petrus, Jaffly Jubili, Robin Lietz, Jesse F. Abrams, Jason Hon, Lukmann H. Alen, Nicholas T. K. Ting, George T. N. Wong, Ling T. Tchin, Nelson J. C. Bijack, Stephanie Kramer‐Schadt, Andreas Wilting, Rahel Sollmann

**Affiliations:** ^1^ Department of Ecological Dynamics Leibniz Institute for Zoo and Wildlife Research Berlin Germany; ^2^ Institute of Ecology, Technische Universität Berlin Berlin Germany; ^3^ Panthera Malaysia Kuala Lumpur Malaysia; ^4^ Global Systems Institute and Institute of Data Science and Artificial Intelligence, University of Exeter Exeter UK; ^5^ WWF‐Malaysia Kuching Malaysia; ^6^ Ta Ann Holdings Berhad Sibu Malaysia

**Keywords:** community occupancy, industrial tree plantations, Malaysia, sustainable forest management

## Abstract

To offset the declining timber supply by shifting towards more sustainable forestry practices, industrial tree plantations are expanding in tropical production forests. The conversion of natural forests to tree plantation is generally associated with loss of biodiversity and shifts towards more generalist and disturbance tolerant communities, but effects of mixed‐landuse landscapes integrating natural and plantation forests remain little understood. Using camera traps, we surveyed the medium‐to‐large bodied terrestrial wildlife community across two mixed‐landuse forest management areas in Sarawak, Malaysia Borneo which include areas dedicated to logging of natural forests and adjacent planted *Acacia* forests. We analyzed data from a 25‐wildlife species community using a Bayesian community occupancy model to assess species richness and species‐specific occurrence responses to *Acacia* plantations at a broad scale, and to remote‐sensed local habitat conditions within the different forest landuse types. All species were estimated to occur in both landuse types, but species‐level percent area occupied and predicted average local species richness were slightly higher in the natural forest management areas compared to licensed planted forest management areas. Similarly, occupancy‐based species diversity profiles and defaunation indices for both a full community and only threatened and endemic species suggested the diversity and occurrence were slightly higher in the natural forest management areas. At the local scale, forest quality was the most prominent predictor of species occurrence. These associations with forest quality varied among species but were predominantly positive. Our results highlight the ability of a mixed‐landuse landscape with small‐scale *Acacia* plantations embedded in natural forests to retain terrestrial wildlife communities while providing an alternate source of timber. Nonetheless, there was a tendency towards reduced biodiversity in planted forests, which would likely be more pronounced in plantations that are larger or embedded in a less natural matrix.

## Graphical abstract

Industrial tree plantations are expanding to offset declining timber supply by shifting towards more sustainable forestry practices and to meet global demand for timber products. Using camera traps, we surveyed the medium‐to‐large bodied terrestrial wildlife community across two mixed‐landuse forest management areas in Sarawak, Malaysia Borneo which include areas dedicated to logging of natural forests and adjacent planted *Acacia* forests. Our results highlight the ability of a mixed‐landuse landscape with small‐scale *Acacia* plantations embedded in natural forests to retain terrestrial wildlife communities while providing an alternate source of timber.

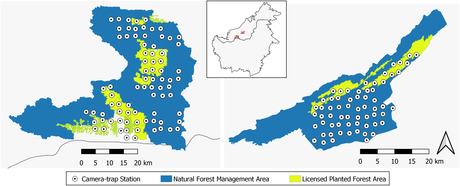

## 1 INTRODUCTION

Expansion of agriculture, tree plantations, and logging activity are primary drivers of the loss and degradation of suitable habitat for tropical wildlife communities (Bradshaw et al., 2009; Giam, 2017). Reduced impact logging and sustainable forest management practices are essential for mitigating species biodiversity loss and maintaining overall ecosystem function and the provision of ecosystem services in tropical forests (FAO, 2018; Sodhi et al., 2010; Struebig et al., 2015). As management of tropical production forests looks towards more sustainable practices, lower timber extraction volumes from logging activities result in reduced financial revenue. Therefore, logging companies have started to convert areas previously under natural forest management into industrial tree plantations dominated by fast growing tree species such as *Acacia mangium* to fuel the global demand for timber products and to increase their financial revenues (FAO, 2010; Giman et al., 2007). Such plantations are now expanding rapidly in Southeast Asia (Mang & Brodie, 2015; Yaap et al., 2016).

As of 2010, The Southeast Asian island of Borneo had already lost about half of its tropical forest cover; of the remaining forest, only 53.8% was classified as intact forest; however, 42% of this forest fell within areas designated for production (Gaveau et al., 2014). Studies assessing the impact of different logging regimes on biodiversity in Borneo largely showed that many species persist in logged landscapes particularly if managed sustainably (Bohnett et al., 2022; Brodie et al., 2015; Meijaard et al., 2005; Sollmann et al., 2017; Wong & Linkie, 2013). At the same time, large areas of Borneo have already been rapidly converted to industrial tree plantations (Chan, 1998; McShea et al., 2009; Rautner, 2005; Reynolds et al., 2011) and further expansion is expected (Gaveau et al., 2016). This is of particular conservation concern as the conversion of natural forests to industrial tree plantations has been shown to result in biodiversity loss and shifts in wildlife community composition to more generalist and disturbance tolerant species (Mang & Brodie, 2015; McShea et al., 2009; Nasi et al., 2008; Peh et al., 2006; Phommexay et al., 2011; Styring et al., 2011; Wanger et al., 2010).

The ecological impacts of a shift from natural forests to industrial tree plantation depend on a variety of factors such as plantation or species characteristics. There is evidence, for example, that the richness of native terrestrial mammal species in *Acacia* plantations can be similar to species richness within the intact or secondary forests, particularly in older plantations (Mang & Brodie, 2015; McShea et al., 2009; Meijaard et al., 2010). Additionally, the impact of tree plantations on individual species varies across niches and behaviors. For instance, herbivores may benefit from differences in forest structure and vegetation cover, while frugivorous species may be negatively impacted as *Acacia* do not produce any fruits or seed crops (McShea et al., 2009). Moreover, the environmental context of plantation forests likely influences their impacts. Patches of remnant forests embedded in a plantation‐dominated landscape can serve as sources of wildlife for plantation forests, thus allowing it to retain species richness similar to natural, unlogged forests (McShea et al., 2009; Ng et al., 2021; Yaap et al., 2016), but this capacity likely depends on the extent and spatial arrangement of remnant forest patches. Additionally, forest management activities are associated with increased road infrastructure, increasing accessibility for other human activities such as hunting (Brodie et al., 2015; Clements et al., 2014), which in turn can affect wildlife communities. Overall, the specific ecological impacts of plantation forests on wildlife remain little understood (Bennett et al., 2000; Brockerhoff et al., 2008; McShea et al., 2009; Meijaard et al., 2005). With industrial plantations projected to continue expanding on Borneo and elsewhere, there is an urgent need to better understand the value of different land‐use regimes towards conservation of tropical mammals in order to mitigate their impacts on biodiversity.

Our objective was to assess the response of medium‐to‐large bodied terrestrial wildlife to mixed land‐use forests in Sarawak, Malaysian Borneo. Combining camera‐trapping with community occupancy modeling, we investigated how species‐specific occupancy and community‐wide diversity patterns were affected by industrial *Acacia* plantations embedded within natural production forests in two logging concessions. Owing to their reduced habitat complexity and resource availability (Brockerhoff et al., 2008), we expect species occupancy to be lower in plantation forests, on average; additionally, on a smaller scale, we expect associations with local forest quality to vary among species due to different levels of disturbance tolerance and different ecological requirements (e.g., herbivores vs frugivores). Finally, owing to the mobility of the focal species group (e.g., Yaap et al., 2016), we expect overall richness to be similar in both forest types, but diversity to be lower in plantation forests as more disturbance‐tolerant species dominate the community. Whereas previous studies have focused on the role of forest remnants in landscapes dominated by plantations, our study provides complementary insight into the effect of small‐scale plantations embedded in natural forests, thus furthering our understanding of the ecological impacts of mixed‐landuse landscapes on the terrestrial wildlife communities in tropical forests.

## 2 MATERIALS AND METHODS

### 2.1 Study sites

**FIGURE 1:**
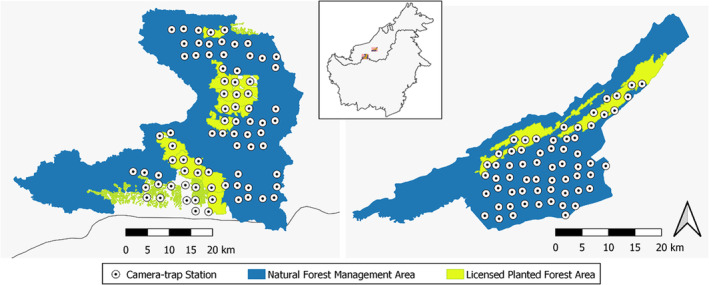
Map of camera‐trap stations and licensed boundaries for forestry activities within Pasin Forest management unit and LPF/0010 (left) and Raplex Forest management unit and LPF/0040 (right), located in Sarawak, Malaysian Borneo (inset)

The two study sites, Pasin Forest Management Unit (Pasin FMU), Raplex Forest Management Unit (Raplex FMU), and neighboring License areas for Planted Forests (license numbers LPF/0010 and LPF/0040 respectively) are located in South‐central Sarawak, Malaysian Borneo, along the Rajang River (Figure 1). Pasin FMU (1324 km^2^) and Raplex FMU (640 km^2^) are predominantly mixed dipterocarp forests with typical equatorial rainforest climate (temperatures ranging between 22–35°C and >2000 mm annual rainfall, see Maiwald et al., 2020) and a rugged landscape with mean elevation (± SD) of 261.77 ± 154.37 m above sea level (between 4.21–1105.80 m) in Pasin FMU and 265.45 ± 171.46 m above sea level (between 33.57–930.22 m) in Raplex FMU. Both forest concessions are managed by a private logging company, Ta Ann Holdings Berhad, which (during the duration of this study) was actively pursuing certification for sustainable management. This involves a transition from conventional selective logging to reduced impact logging (RIL) practices, whereby the placement of logging roads and skid trails and harvesting methods are adjusted to reduce forest disturbance (Enters & Durst, 2002; Putz et al., 2001). Pasin FMU is divided into 25 timber harvesting annual coupes and has undergone continued logging activity since 1985. Raplex FMU is divided into 20 timber harvesting annual coupes and was initially licensed for logging in 1977. Both forest management units are adjacent to (and/or geographically encompass) areas under License for Planted Forest (*Acacia)*, with licensed area LPF/0010 adjacent to Pasin FMU representing 16.4% of the study site and licensed area LPF/0040 adjacent to Raplex FMU representing 13.1% of the total study site. Though the licensed areas are dedicated to planted *Acacia*, large areas of natural forests are still present (>50% of the LPF areas). Local communities that practice shifting agriculture are located along the major rivers running through the forest sites. Residents live mainly as subsistence farmers, work in nearby timber and plantation camps, and are involved in hunting to some degree.

### 2.2 Wildlife survey

We surveyed Pasin FMU and LPF/0010 (henceforth, just Pasin FMU) between the months of January–August 2018 and Raplex FMU and LPF/0040 (henceforth, Raplex FMU) between the months of April–August 2019 using camera traps. A total of 142 stations (79 in Pasin FMU and 63 in Raplex FMU) were set spaced at approximately 3‐km intervals. Of the 142 camera‐trap stations, 96 stations were set within areas dedicated to natural forest management while 43 were placed within the licensed planted forest areas. Three stations were set within natural forest adjacent to Pasin FMU and LPF/0010 boundaries, but environmental conditions at these sites were within the range of other locations in Pasin FMU and we, therefore, included them in the NFM category. At each station, 2 different cameras (a combination of one Reconyx PC850 HyperFire Pro White Flash with one Covert Illuminator or PantheraCam V3) were deployed <5 m apart, 30–45 cm above ground, and each oriented towards either different sections of the same or two separate logging roads or animal trails. We programmed cameras to take 3 consecutive images during each detection. We cleared vegetation to reduce false triggering of cameras and retrieved cameras after a minimum of 60 days of operation.

We identified animals in images to species, with muntjacs (Bornean yellow muntjac *Muntiacus antherodes* and Southern red muntjac *M. muntjac*), mongooses (collared mongoose *Herpestes semitorquatus* and short‐tailed mongoose *H. brachyurus*), and mousedeer (greater mousedeer *Tragulus napu* and lesser mousedeer *T. kanchil*) identified only to genus due to similarities in morphology and ecology between sister species. Small mammals (rat, squirrel, and tree shrew species) were identified according to broader taxonomic groups due to difficulty in discerning species in photos. We used the package camtrapR version 0.99.5 (Niedballa et al., 2016) in program R version 4.0.3 (R Core Team, 2020) to organize and build a record database from all cameras. For each station, we combined all records taken by both cameras.

### 2.3 Habitat covariates

Though forest landuse type captures broad differences in ecological conditions, the categorization of stations based on license boundaries alone would ignore within‐category variation in ecological conditions that may be important for terrestrial wildlife. To characterize the habitat conditions within each forest management unit and at each camera‐trap station, we initially produced and extracted seven GIS‐based covariates (see Table S1 for a summary of covariates). To assess the response of the terrestrial wildlife community to forest conditions within mixed‐landuse landscapes at a fine scale, we used the Structural Conditions Index (SCI) and percent canopy cover from previously published datasets (30‐m resolution, Hansen et al., 2019) as measures of forest disturbance and degradation. The SCI is a weighted value calculated by combining information on canopy cover, canopy height, and time since forest lost; values range between 1 and 18, with higher values representing less degraded forests (see Hansen et al., 2019). Beyond these predictors of main interest to the present study, elevation and terrain ruggedness have the potential to influence species richness and distributions (Amatulli et al., 2018); to account for this, we extracted the elevation at each camera station and estimated the terrain ruggedness index (TRI) from a digital elevation model (30‐m resolution, National Aeronautics and Space Administration Shuttle Radar Topography Mission) using the “terrain” function from the R‐package raster version 3.1‐5 (Hijmans, 2020) to calculate the differences in slope within a 3 × 3 and 7 × 7 pixel neighborhood.

Aside from forest and landscape characteristics, hunting pressure can also be a prominent driver of wildlife distribution (Benitez‐Lopez et al., 2019). Forest management activities are often accompanied by road infrastructure which increases access and, thus, the opportunity for hunting (Bennett et al., 2000; Brodie et al., 2015; Robinson et al., 1999). To account for the potential effects of hunting, village density and distance to nearest access were considered as proxies for hunting pressure. Locations of licensed area boundaries, villages, roads, and rivers were provided by Ta Ann Holdings Bhd., and we used the “distance to nearest” analysis tool in QGIS 3.10 (QGIS, 2020) to record the distance of each station from the nearest point of access (logging road, skid trail, or large river). Additionally, we created a heatmap in QGIS with the default quartic kernel decay function at a 15‐km radius around each village point to calculate village density values (Tilker et al., 2020). Higher density values at a camera‐trap station represent closer proximity to villages and thus, relatively higher hunting pressure.

Habitat covariates were scaled (mean of zero and variance of 1) and tested for correlations by calculating Spearman Rank Correlation coefficients (Figure S1); covariates were considered substantially correlated if the absolute value of the coefficient was >0.7 (Dormann et al., 2013). For correlated covariates, we ran single covariate community occupancy models and retained the covariate for which more species showed strong associations (see *Data analysis*). Our final selection of habitat covariates to represent the conditions around each camera‐trap station included SCI, distance to the nearest point of access, elevation, and terrain ruggedness at a 3 × 3 pixel neighborhood.

### 2.4 Data analysis

In order to assess the influence of covariates on species occurrence within the FMUs, we utilized Bayesian community occupancy models (e.g., Sollmann et al., 2017). Occupancy models use repeated species‐level binary detection/non‐detection data collected across multiple sampling locations to estimate species occurrence probability (and its response to covariates) while accounting for imperfect and varying species detection (MacKenzie et al., 2005). By jointly analyzing data from multiple species, community occupancy models increase the precision of parameter estimates for rare species by ‘borrowing’ information from data‐rich species, assuming that species‐specific parameters come from a common parametric distribution, governed by community parameters (Royle & Dorazio, 2008). Because detection/non‐detection data is comparatively easy to collect with camera‐traps, even for rare and cryptic species in challenging terrain, fitting community occupancy models to camera‐trap data has become a widely used approach to studying the spatial ecology of terrestrial vertebrates (Bajaru et al., 2020; Devarajan et al., 2020; Rahman et al., 2021).

To transform raw camera records into a format suitable for occupancy modeling, we condensed data into 5‐day sampling occasions, determining for each station and occasion whether a species had been detected (1) or not (0). We modeled detection probability as having a species‐specific random intercept with site‐specific hyperparameters to account for potential differences in detection due to surveying each FMU site at different times. Additionally, we accounted for varying survey efforts due to malfunctioning camera‐traps by including the number of days each camera at a station was functional within a 5‐day occasion (i.e. effort = 10 if both cameras were functional during the whole occasion) as a fixed effect, and the effect of camera placement by including whether cameras were set on‐ or off‐road as a random (species‐level) effect on detection. We modeled occupancy probability as having a species‐specific random intercept and included natural‐ and plantation‐forest specific hyperparameters to allow for different baseline occupancy levels in areas of natural forest management (NFM) and licensed planted *Acacia* forest (LPF). Further, to assess the fine scale habitat associations within the different landuse types, our community model included species‐specific effects of the four habitat covariates (SCI, distance to nearest access, elevation, and TRI) on occupancy.

We implemented the model in a Bayesian framework using the R‐package *nimble* version 0.12.1 (de Valpine et al., 2017, 2021). We ran three parallel Markov chains with 300,000 iterations each, of which we discarded the first 50,000 as burn‐in and further thinned the remaining iterations by 20. We assessed chain convergence using the R‐hat statistic (Gelman et al., 2004); all chains showed *R*‐hat values <1.1, indicating convergence. We report results as posterior mean, standard deviation, and the 95% and 75% Bayesian credible intervals (95% BCI according to the 2.5% and 97.5% percentiles of posterior distribution, and 75% BCI according to the 12.5% and 87.5% percentiles). We consider a coefficient to have strong support if the 95% BCI did not overlap zero and moderate support if the posterior 75% BCI did not overlap zero. We used the parameter estimates from the community model to predict the occupancy probability for each of the species for all 30 x 30 m grid cells comprising the total study landscape (2,258,965 cells in NFM and 409,888 cells in LPF). We then generated a Bernoulli random variable for each cell based on the corresponding predicted occupancy probability to determine if a cell was occupied by a given species (i.e., had a generated value of 1), and we calculate the percent of cells occupied for each species (percentage of area occupied, PAO) separately for NFM and LPF areas. We performed predictions for every 20th posterior sample of the parameters and report the mean over these repeats with standard deviation and 95% BCI.

To further compare the species communities between NFM and LPF, we calculated occupancy‐based defaunation indices and diversity profiles based on mean predicted occupancy. The defaunation index is a modification of the Bray‐Curtis index quantifying the dissimilarity between a (typically less disturbed) reference and a focal assemblage (Giacomini & Galetti, 2014); the index is typically based on species abundance in each assemblage, but can also be calculated based on mean predicted occupancy (Tilker et al., 2020). We use NFM as the reference assemblage because it is less disturbed relative to LPF areas. Dissimilarity values can range between −1 and 1, where negative values indicate a more complete community in LPF (i.e, species have gained in occupancy) compared to NFM, 0 indicates no difference in assemblages between the two different forest management areas, and positive values indicate less complete community in LPF (i.e., species have lost occupancy) compared to NFM areas. We expect a positive index, indicating that at least some species lose part of their distribution in LPF compared to the NFM areas. Similarly, we used mean predicted occupancy to generate occupancy‐based diversity profiles and compare the differences in predicted species diversity between NFM and LPF (Abrams et al., 2021). Diversity profiles are a plotted series of diversity estimates (specifically, Hill numbers), including multiple common diversity indices (e.g., species richness, Shannon and Simpson diversity), along a gradient *q* that quantifies the impact of rare species on diversity (Leinster & Cobbold, 2012). At *q* = 0, all species contribute to diversity equally regardless of their rareness, and the corresponding diversity value equals species richness. As *q* increases, rare species contribute less to diversity. The shape of the diversity profile informs us about the richness and evenness of a community: a more steeply declining profile indicates a community that is less even (i.e., more dominated by a few common species). Finally, to assess the effects of shifting from NFM to LPF for species of particular conservation concern, we generate a separate defaunation index and diversity profile for endemic species and those listed as Near Threatened or higher on *The IUCN Red List of Threatened Species*.

## 3 RESULTS

We collected 10,186 independent records of 44 species (36 mammals, 5 birds, and 3 reptiles) over the course of 13,567 trap nights. Of these records, we excluded all species with <5 detections from the analysis. Additionally, we excluded all arboreal, reptile, and small mammal species, as they are poorly sampled by our camera trap setup. Furthermore, we excluded wide ranging species to maintain the assumption of sampling location independence in occupancy modeling. Our final species list consisted of 25 medium‐to‐large bodied, terrestrial species (22 mammals and 3 birds) with 15 species considered endemic to Borneo and/or globally threatened on *The IUCN Red List of Threatened Species*. All but one species were recorded in both forest types, with Hose's civet *Diplogale hosei* only recorded in NFM. Additionally, Hose's civet was only recorded in Pasin FMU while Asian small‐clawed otter *Aonyx cinereus* and common palm civet *Paradoxurus hermaphroditus* were only recorded in Raplex FMU. See Table S2 for a summary of species detections.

Natural forest had a significant effect on the occurrence of two species (one positive, one negative); for all other species the estimated forest type specific intercepts were similar (see Figure S2). Species‐specific occurrence responses to SCI, distance to the nearest access point, elevation, and TRI varied in direction and strength (Figure 3). We found a strong positive association between occupancy probability and SCI for 5 species and moderate positive associations for 3 species; at the community level, occupancy probability had a moderate positive association with SCI. Only one species had a moderate positive association with distance to the nearest access point. Two species showed a moderate association with elevation (1 positive and 1 negative), and three species had a moderate negative association with TRI. The associations with distance to nearest access, elevation, and TRI were weak at the community level. Camera‐trap effort had a weak, positive effect on detection probability (0.20 ± 0.01). Community mean detection probability was slightly higher in the Raplex FMU (−4.93 ± 0.37 on the logit scale) than in the Pasin FMU (−5.21 ± 0.42). The association with on‐road camera setup at the community level was positive but weak, but the detection probability of one species had a strong positive association with on‐road camera placement.

**FIGURE 2:**
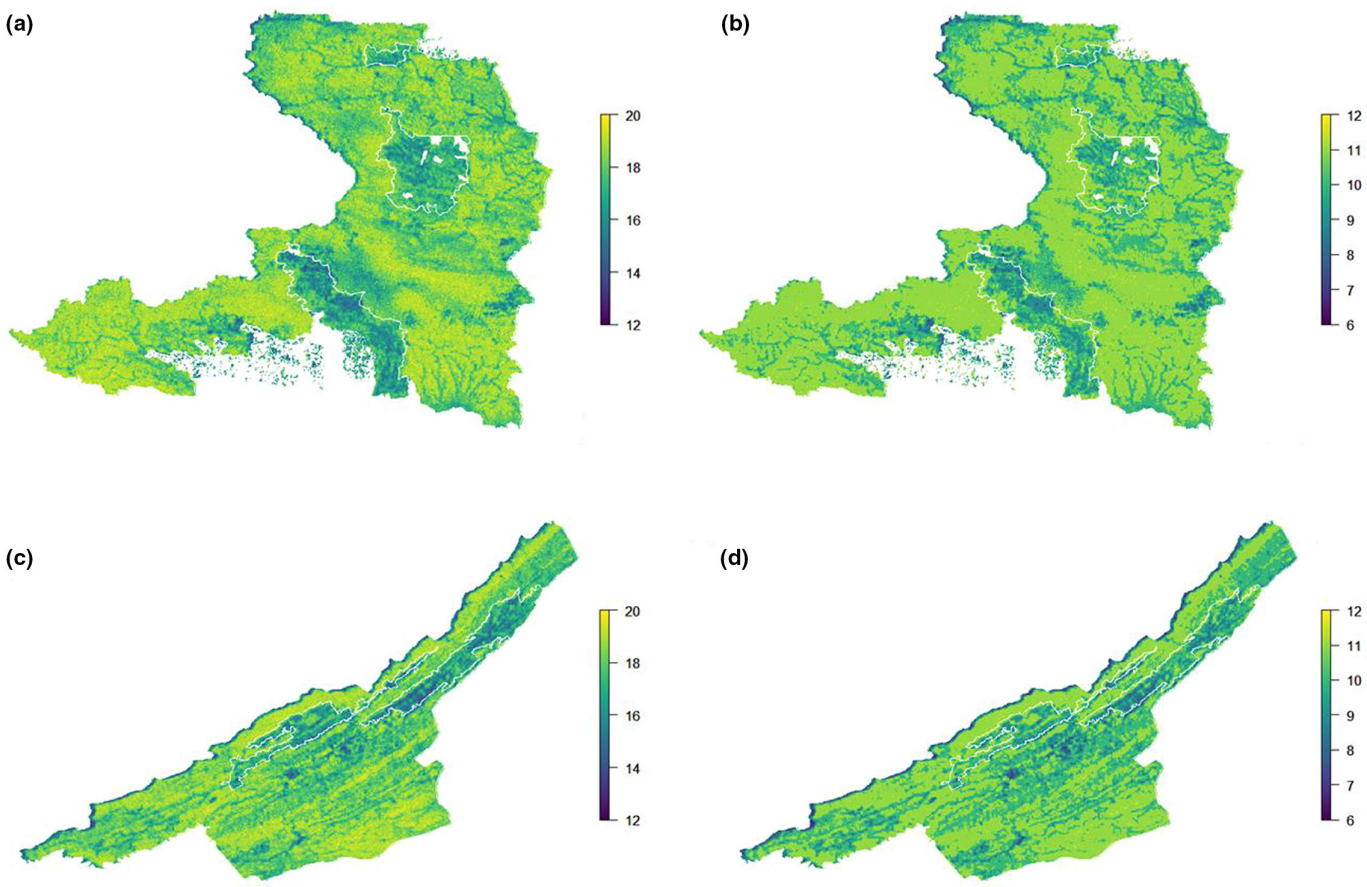
Predicted species richness per 30 × 30‐m cell across two mixed‐landuse forest management units in Central Sarawak, Malaysian Borneo, based on community occupancy model fit to camera data. Top (A and B): Maps of the distribution of predicted species richness across Pasin Forest management unit for (a) full community of 25 medium‐to‐large terrestrial wildlife species and (b) for 15 threatened and endemic species. Bottom (C and D): Maps of the distribution of predicted species richness across Raplex Forest management unit for (c) full community of 25 medium‐to‐large terrestrial wildlife species and (d) for 15 threatened and endemic species. White lines represent boundaries of licensed *acacia* plantation areas, these are embedded within naturally managed forest.

Based on area‐wide predictions, all species were expected to occur in both forest types. The NFM areas showed higher predicted average species richness (18.18 ± 0.92) per 30 × 30‐m cell compared to LPF (16.85 ± 1.04; Figure 2a, c). Similarly, the predicted average richness of the subset of globally threatened and endemic species per 30 × 30‐m cell was marginally higher in NFM (10.51 ± 0.72) compared to LPF (10.01 ± 0.82; Figure 2b, d). The predicted PAO of the 25 species differed between the two forest management types, with 14 species having larger PAOs in NFM than LPF (Figure S3). However, most differences were small and the 95% confidence intervals for mean PAO overlapped between the two forest types for all species.

**FIGURE 3:**
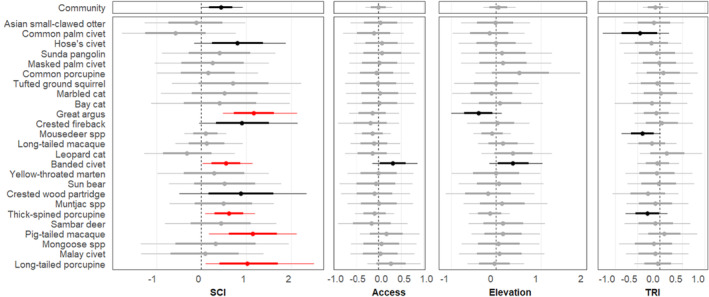
Model coefficients (mean and Bayesian credible intervals, BCI) for the effects of structural conditions index (SCI), distance to nearest access point (access), elevation, and terrain ruggedness (TRI) on the occupancy probabilities of 25 medium‐to‐large terrestrial wildlife species, estimated using a community occupancy model fit to camera‐trap data from two forest management units in Sarawak, Malaysian Borneo. Thin error bars represent the 95% BCI and thick error bars represent the 75% BCI. Red dots/bars indicate strong associations between a covariate and occupancy (95% BCI not overlapping zero), black dots/bars represent moderate associations (75% BCI not overlapping zero), and gray represents weak association.

**FIGURE 4:**
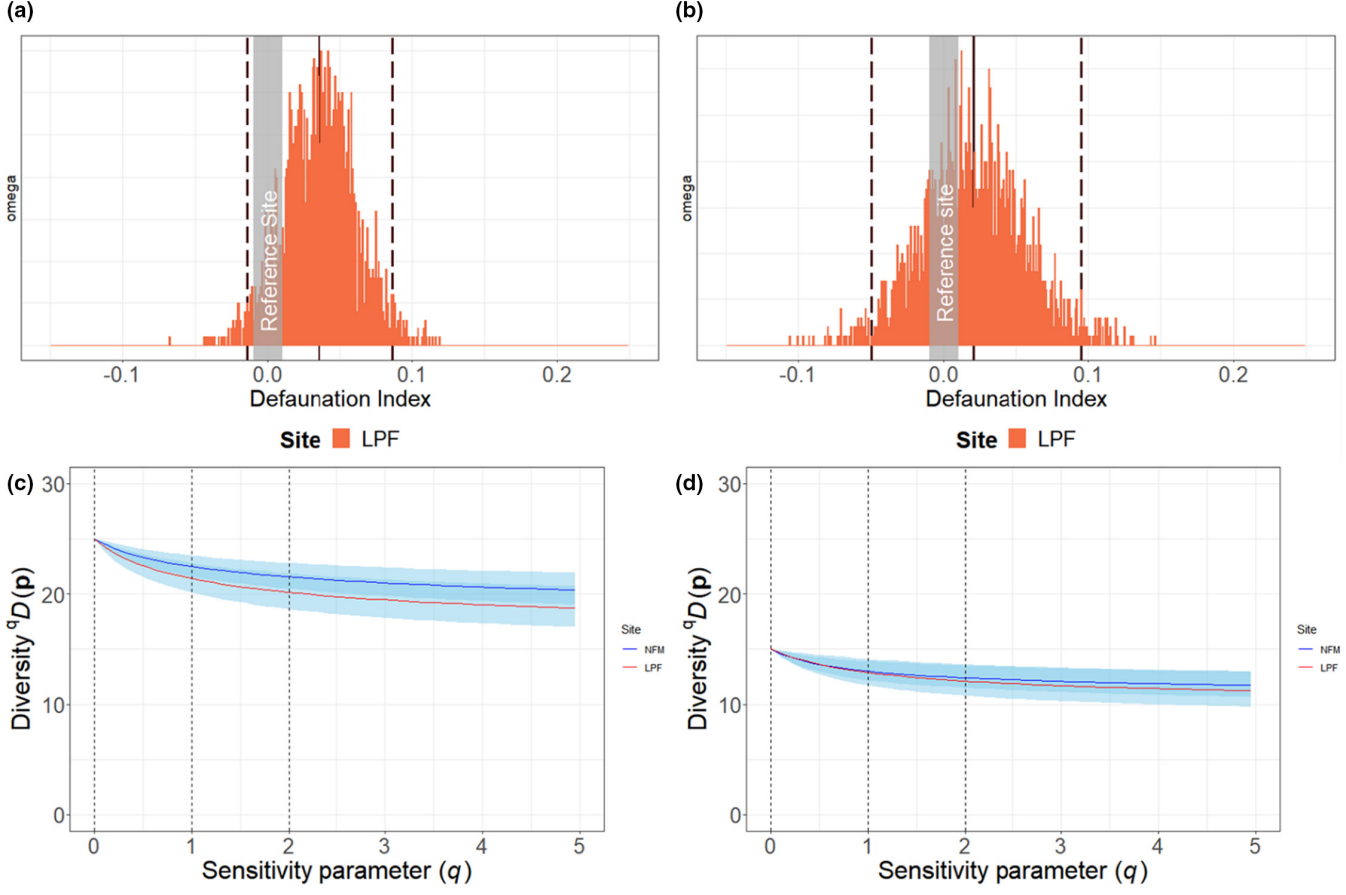
Top (A and B): Occupancy‐based species defaunation index, calculated from community occupancy model predictions, for the full community of 25 medium to large terrestrial wildlife species (a) and for 15 threatened and/or endemic species (b) in licensed planted forest (LPF), based on camera trap data from two logging concessions in Sarawak, Malaysian Borneo. The natural forest management (NFM) area is used as a reference site (zero defaunation). Solid line represents mean values; dotted lines represent the 95% Bayesian credible intervals, and histogram shows posterior distribution of the defaunation index. Bottom (C and D): Species diversity profiles calculated from community occupancy model predictions, for NMF and LPF areas for the full community of 25 species (c) and for 15 threatened and/or endemic species (d), with standard deviations (light blue shading). Includes three diversity indices (vertical dotted lines): Species richness (*q* = 0), Shannon index (*q* = 1) and Simpson index (*q* = 2).

For the full 25‐species list, the occupancy‐based defaunation index value was positive but small (0.05 ± 0.03) indicating a slightly lower mean predicted occupancy for the community and species within LPF areas relative to NFM (Figure 4a). The defaunation index value for threatened and endemic species was also positive and slightly smaller (0.04 ± 0.04, Figure 4b). Occupancy‐based diversity profiles for both the full community and threatened and endemic species declined more quickly in LPF relative to NFM, indicating less even communities (Figure 4c, d), but Bayesian credible intervals for both profiles overlapped. Curves for both forest management types were relatively flat, suggesting similar and low sensitivity to the occurrence of rare species.

For the full 25‐species list, the occupancy‐based defaunation index value was positive but small (0.05 ± 0.03) indicating a slightly higher mean predicted occupancy for the community and species within NFM areas relative to LPF (Figure 4a). The defaunation index value for threatened and endemic species was also positive and slightly smaller (0.04 ± 0.04, Figure 4b). Occupancy‐based diversity profiles for both the full community and threatened and endemic species declined more quickly in LPF relative to NFM, indicating less even communities (Figure 4c, d), but Bayesian credible intervals for both profiles overlapped. Curves for both forest management types were relatively flat, suggesting similar and low sensitivity to the occurrence of rare species.

## 4 DISCUSSION

Our study in two mixed‐landuse forest management areas in Sarawak, Malaysian Borneo, confirmed our prediction that small‐scale commercial plantations of *Acacia mangium* (License for Planted Forest, LPF) adjacent to natural forest management (NFM) areas in a mixed‐landuse landscape were used by the same terrestrial wildlife species as the neighboring natural forests. As expected, species‐level percent area occupied was smaller in LPF for most species, and diversity was lower, indicating negative effects of LPF on the terrestrial wildlife community, but differences were generally small. The importance of forest habitat integrity for this community was more pronounced on a smaller spatial scale. The structural conditions index (SCI) was the most important predictor of occupancy probability both for the community and for individual species. Even though as expected strength and direction of the effect varied, all moderate‐to‐strong associations were positive. The SCI was, on average, lower in LPF than in NFM compartments (Table S1), further indicating the negative effects of plantation forests on habitat quality. But SCI was highly variable within each forest management type, which likely contributed to the small differences in species occurrence we observed between forest types. This variability likely stems from the presence of natural forest and/or age of the planted forest mosaics inside LPF boundaries. Combined with the importance of SCI for many species, this suggests that local negative effects of LPF (due to lower SCI) on the terrestrial wildlife community may be stronger than our comparison of the two forest types based on license boundaries indicates (as LPF boundaries also contain non‐planted forests). Nonetheless, at the scale of these forest concessions, our results highlight the ability of a mixed‐landuse landscape incorporating a mosaic of small‐scale planted forests to retain terrestrial wildlife communities present in the naturally managed forest while providing an alternate source of timber and revenue.

During our survey, we recorded a diverse species assemblage including two little known carnivore species endemic to Borneo, the Hose's civet *Diplogale hosei* and Bay cat *Catopuma badia*, and several globally threatened species such as the critically endangered Sunda pangolin *Manis javanica*. The list of mammals recorded during our survey was similar compared to camera‐trap surveys of other production forests (Maiwald et al., 2020–34 mammal species, Sollmann et al., 2017–28 mammal species) and protected primary forests (Mohd‐Azlan & Engkamat, 2013–26 terrestrial mammals and birds with much lower sampling effort) on Borneo, suggesting that the study areas still harbor a full terrestrial mammal assemblage. As expected, within this landscape, both estimated richness‐per‐cell and richness across the two different landuse areas were similar but slightly higher in NFM. This is consistent with other studies involving *Acacia* plantation areas that also showed similar estimates of species richness between forest plantations and secondary forests (Mang & Brodie, 2015; Ng et al., 2021). Within our study sites, this could be due to the presence of natural and secondary forests within the licensed areas dedicated to *Acacia* plantations, as well as the low percentage of overall area covered by LPF. Further, many species in the focal community are highly mobile (even though we excluded the most wide‐ranging species from analysis) and could readily use both forest types, even if they depended on, or preferred, natural forest. Yue et al. (2015), for example, showed that species richness in oil palm plantations dropped quickly with increasing distance from forest, suggesting that animals may venture into the plantations (and thus, contribute to richness estimates there) but may depend on forest habitat for their persistence. Similar to richness, dissimilarity indices and diversity profiles (which take into account species prevalence and, for the former, species identity) only showed minor differences between LPF and NFM, which were slightly bigger for threatened and/or endemic species. The positive defaunation indices suggested some loss in species percent area occupied in LPF relative to NMF and the diversity profiles suggested the community was slightly more even and thus, diverse, in NMF. Thus, across all measures there seems to be a trend towards lower diversity in LPF. The weakness of this trend is likely due to the small scale of, and extensive presence of natural forests within, LPF in the study sites. Other camera‐trap studies in landscapes dominated by plantation forests reported stronger reductions in observed and estimated richness within areas of commercial tree plantations compared to forest buffers and secondary forests (McShea et al., 2009; Yaap et al., 2016), highlighting that the effects of plantation forests are context‐dependent. To better assess the risks to biodiversity but also the conservation potential of these plantation forests within a mixed‐landuse landscape, it is important to further investigate how the amount and spatial configuration of both natural forest areas and commercial plantations within mixed‐use landscapes modulate plantation effects on wildlife communities.

Observed patterns in richness and diversity were also reflected on the species level where many species in our community occupied larger areas within the NFM, though the differences in PAO for most species were relatively small. Only for three species (crested partridge *Rollulus rouloul*, common palm civet *Paradoxurus hermaphroditus*, and banded civet *Hemigalus derbyanus*), the mean estimate of PAO in LPF was lower than the 2.5th percentile of the corresponding estimate of PAO in NFM, and for one species (Hose's civet) the mean estimate of PAO in LPF was higher than the 97.5th percentile of the corresponding estimate of PAO in NFM. For crested partridge and banded civet, these patterns are consistent with other studies that found these species to be associated with less disturbed forests (Nijam, 1998; Ross et al., 2016; Savini et al., 2021; Winarni et al., 2009). Though common palm civet are considered to be disturbance tolerant habitat generalists, a smaller estimated PAO in LPF could be due to limited food availability for frugivorous species in plantations of *Acacia mangium* (Nakashima et al., 2010). However, for Hose's civet, the PAO result contrasts with previous studies which suggest negative effects of forest disturbance on occurrence (Mathai et al., 2016, 2019), as well as its strong positive association with forest quality (measured by SCI) in the present study. Similarly, we found that in spite of a strong positive association with SCI, both Southern pig‐tailed macaque *Macaca nemestrina* and great argus *Argusianus argus* had higher estimated PAO in LPF areas. These results can be attributed to differences in baseline occupancy between the respective land use areas and suggest that while these species are associated with more/less disturbed forests on a small scale, unmeasured characteristics of plantation forests also affect their occurrence.

The SCI was the most prominent habitat covariate in our community model, with moderate to strong positive effects on 8 species as well as the entire community. In general, forest quality is an important predictor of species occurrence, particularly in production forests experiencing moderate levels of disturbance (Sollmann et al., 2017; Tilker et al., 2019). As we predicted, the associations with forest quality varied among different species but were predominantly positive. The finding that all 3 ground‐dwelling birds, crested partridge, great argus, and Bornean crested fireback *Lophura ignita*, had positive associations with forest quality is consistent with studies which suggest associations with primary and less disturbed forests (Savini et al., 2021). All three species are considered globally threatened, and their occurrence in the production landscapes highlights the value of mixed‐landuse landscapes for conserving species (Grainger et al., 2018). Positive associations with SCI for the largely frugivorous southern pig‐tailed macaques also matched prior knowledge suggesting the species is forest‐dependent and prefers high‐quality forest (e.g. Sollmann et al., 2017), though it can also occupy logged forests and tree plantations (e.g. Ruppert et al., 2018). Little is known about the ecology of the remaining two species with moderate/strong positive association with forest quality, thick‐spined porcupine *Hystrix crassispinis* and long‐tailed porcupine *Trichys fasciculata*. Both porcupine species are known to occupy degraded habitats (Cassola, 2016; Lunde et al., 2016), though they may nonetheless prefer high‐quality forest when they have access to it.

Though elevation and terrain ruggedness as characteristics of landscapes are known to influence species distributions, our results showed weak associations with community occupancy, with very few moderate associations. This is perhaps due to the fact that our sampling did not extend into altitudes where lowland rainforest is replaced with more montane habitat. The habitat type was consistent across the range of elevations and terrain ruggedness represented in our data (Table S1). Similarly, community and species‐specific occupancy was mostly weakly associated with distance to the nearest logging road or larger river (only two species showed moderate associations), which was included in our community model as a proxy for hunting pressure. Hunting is a major driver of species declines across the tropics (Benitez‐Lopez et al., 2017; Tilker et al.,  2019; Tilker et al., 2020). The lack of any strong associations with distance to the nearest access point in our study could indicate that hunting activity did not occur at high enough levels to influence wildlife community and species occurrence, particularly as there is a ban on hunting in the licensed areas and a restriction of entry by outsiders (hunters) using logging roads into the concessions. Alternatively, as hunting pressure is difficult to quantify (e.g. Sollmann et al., 2017; Tilker et al., 2020), a more accurate or direct measurement of hunting pressure may be necessary to reveal its effects on wildlife occurrence.

Whereas we consider the relatively small proportion of LPF in our study areas and the extensive presence of natural forests within LPF license boundaries as the main reasons for the observed weak effects of LPF on terrestrial wildlife occurrence and diversity, other characteristics of the study likely contributed as well. Our study sites were exclusively mixed‐landuse landscapes, and therefore we cannot infer how plantation forest affects the terrestrial wildlife community compared to the primary forest (Barlow et al., 2007; Giam, 2017), though such a comparison would likely show more pronounced effects. Similarly, our study area does not represent a highly disturbed landscape such as clear‐cut areas or plantation monocultures where species richness and abundance are reduced (Barnes et al., 2017; Pawson et al., 2005). If species occurring within plantation forests depend on the surrounding natural forest habitat (McShea et al., 2009; Yaap et al., 2016), plantations embedded within more disturbed landscapes will likely have much poorer terrestrial wildlife communities. Additionally, during our study, the mosaic forest plantation areas inside the LPF were not undergoing active operations, with planted *Acacia* trees already exceeding 5 years old. This would potentially allow for the terrestrial wildlife community to recover or return after being displaced by the initial stages of forest plantation development. Previous research suggests younger *Acacia* stands harbor lower species diversity (McShea et al., 2009), and older plantations are higher in habitat complexity and heterogeneity (Mang & Brodie, 2015). As a result, the diverse landscape mosaic of the present study may have sufficient forest quality to maintain a full terrestrial wildlife assemblage, a situation that may not be the case in large monocultures, or even mixed‐use landscapes dominated by more disturbed habitats. Further, our study focuses on medium to large terrestrial species, a group that is frequently studied to assess the impacts of forest disturbance (Maiwald et al., 2020; McShea et al., 2009; Ng et al., 2021). Many other taxa, however, experience biodiversity loss within production landscapes including arboreal species (Haysom et al., 2021), birds (Beukema et al., 2007; Peh et al., 2006; Styring et al., 2011; Waltert et al., 2004), and amphibians (Asad et al., 2020; Wanger et al., 2010). It is possible that other communities of wildlife are more affected by LPF than the highly mobile medium‐to‐large terrestrial species. Finally, our data represent a single‐season survey. Repeat surveys following the rotation of logging and plantation activities would reveal how the effects of these measures may change over time, particularly if the management of these landscapes proceeds in line with sustainable forestry guidelines.

Worldwide, large proportions of tropical rainforests are used for timber production. Though biodiversity loss is inevitable with forest loss and disturbance due to forestry activities (Barlow et al., 2007; Giam, 2017), sustainably managed natural forests play a key role in maintaining biodiversity (Brodie et al., 2015; Sollmann et al., 2017). With ongoing tropical deforestation and the concurrent expansion of industrial tree plantations in Southeast Asia (FAO, 2010; Mang & Brodie, 2015; Ng et al., 2021; Yaap et al., 2016) and elsewhere (McEwan et al., 2020), it is urgent to investigate the ecological effects of industrial tree plantations, so that negative impacts can be avoided or mitigated. Our study suggests that in a mixed‐landuse landscape where plantation forest is embedded within a landscape context dominated by naturally managed forest, plantations can harbor similar terrestrial wildlife assemblages as natural production forests. The presence of plantation forests, however, resulted in lower forest quality. Given the high number and wide variety of species (with respect to size, feeding ecology and taxonomy) responding positively to forest quality, increasing the conversion of natural forests to plantations across Borneo is likely to have far‐reaching negative impacts across the island's terrestrial wildlife community. With limited studies and mixed evidence regarding the effect of plantation forests on wildlife (e.g., Mang & Brodie, 2015), further research is needed to assess how plantation forest effects on communities and species are mediated by other factors such as plantation age, size, spatial configuration and surrounding habitat. This would help further increase our understanding of how mixed‐landuse landscapes can be managed to serve as refuges for terrestrial wildlife communities as areas of natural forest are lost to the ever‐present demand for timber products.

## AUTHOR CONTRIBUTIONS

**Seth T. Wong:** Data curation (lead); formal analysis (equal); funding acquisition (supporting); investigation (equal); writing – original draft (equal); writing – review and editing (equal). **Roshan Guharajan:** Conceptualization (supporting); data curation (equal); funding acquisition (equal); investigation (equal); writing – review and editing (supporting). **Azrie Petrus:** Data curation (equal); investigation (equal). **Jaffly Jubili:** Data curation (equal); investigation (equal). **Robin Lietz:** Data curation (supporting); investigation (supporting). **Jesse F. Abrams:** Formal analysis (equal); methodology (equal); software (equal). **Jason Hon:** Conceptualization (supporting); funding acquisition (equal). **Lukmann H. Alen:** Project administration (equal). **Nicholas T. K. Ting:** Conceptualization (supporting); project administration (equal). **George T. N. Wong:** Project administration (equal). **Ling T. Tchin:** Project administration (equal); writing – review and editing (equal). **Nelson J. C. Bijack:** Project administration (supporting). **Stephanie Kramer‐Schadt:** Supervision (equal); writing – review and editing (equal). **Andreas Wilting:** Conceptualization (equal); funding acquisition (equal); supervision (equal); writing – review and editing (equal). **Rahel Sollmann:** Conceptualization (equal); formal analysis (equal); methodology (equal); software (equal); supervision (equal); writing – original draft (equal); writing – review and editing (equal).

## CONFLICT OF INTEREST

The authors declare that they have no conflict of interest.

## Supporting information


Appendix S1

## Data Availability

Input data and model implementation code are available in Dryad: https://doi.org/10.5061/dryad.tqjq2bw30.
