# Supplementary material for: How do terrestrial wildlife communities respond to small‐scale Acacia plantations embedded in harvested tropical forest?
Source: Ecol Evol. 2022 Sep 20;12(9):e9337. doi: 10.1002/ece3.9337 (PMC9486821; doi:10.1002/ece3.9337)

APPENDIX

**Table S1.** **Top:** Mean, standard deviation (SD), minimum (Min), and maximum (Max) values for seven remote sensing covariates calculated and extracted from 142 camera-trap stations distributed across areas of natural and licensed planted forest in two study sites in Borneo, to characterize the habitat characteristics and conditions surrounding each station. All covariates have 30-m resolution. Structural Conditions Index (SCI) and percent canopy cover (Canopy) values at each station were extracted from previously published datasets. Distance to nearest access point (in meters, dAccess) was calculated as the smallest linear distance between a camera-trap station and either a logging road or large river. Village density values (Village) for each station were calculated from a village heat map. Elevation values (in meters) were extracted at the point locations/coordinates of each camera-trap station. Terrain Ruggedness Index (TRI) was calculated for each station for a 3 x 3 pixel neighborhood (TRI 3 x 3) and a 7 x 7 pixel neighborhood (TRI 7 x 7). **Bottom:** Summary of values across the forest management areas for 4 covariates included in the final community occupancy model.

|  |  | **Natural Forest Management** | | | | **Licensed Planted Forest** | | | |
| --- | --- | --- | --- | --- | --- | --- | --- | --- | --- |
|  |  | Mean | SD | Min | Max | Mean | SD | Min | Max |
| **Station** | SCI | 12.81 | 3.04 | 4.86 | 17.94 | 9.49 | 3.78 | 1.56 | 17.80 |
|  | Canopy | 85.05% | 12.43% | 28.74% | 97.00% | 77.75% | 20.85% | 5.66% | 96.24% |
|  | dAccess | 302.60 | 361.69 | 2.25 | 1830.22 | 376.53 | 488.92 | 7.27 | 2645.08 |
|  | Village | 8.60 | 10.31 | 0.00 | 37.02 | 8.90 | 7.09 | 1.70 | 28.99 |
|  | Elevation | 312.77 | 167.98 | 43 | 906 | 285.81 | 126.05 | 86 | 598 |
|  | TRI 3 x 3 | 7.56 | 4.81 | 1.38 | 31.50 | 7.42 | 5.54 | 1.25 | 30.63 |
|  | TRI 7 x 7 | 16.19 | 6.95 | 5.02 | 36.90 | 16.24 | 7.20 | 3.67 | 37.02 |
|  |  |  |  |  |  |  |  |  |  |
| **Landscape** | SCI | 14.85 | 2.55 | 1 | 18 | 12.04 | 3.30 | 1.23 | 17.99 |
|  | dAccess | 611.69 | 600.05 | 1.1E-05 | 4536.16 | 576.12 | 555.92 | 7.0E-04 | 3806.25 |
|  | Elevation | 262.39 | 166.40 | 4.21 | 1105.80 | 266.30 | 114.58 | 55.97 | 823.64 |
|  | TRI 3 x 3 | 8.09 | 3.60 | 0 | 104.56 | 8.07 | 3.21 | 0.47 | 48.93 |

**Figure S1**. Spearman Rank Correlation coefficients between seven covariates considered to model terrestrial mammal occurrence in natural and licensed plantation forest in two study sites in Borneo: Structural Conditions Index (SCI), percent canopy cover (Canopy), distance to nearest access point (Access), village density (Village), elevation (Elevation), Terrain Ruggedness Index (TRI) at a 3 x 3 pixel neighborhood (TRI3x3), and at a 7 x 7 pixel neighborhood (TRI7x7).


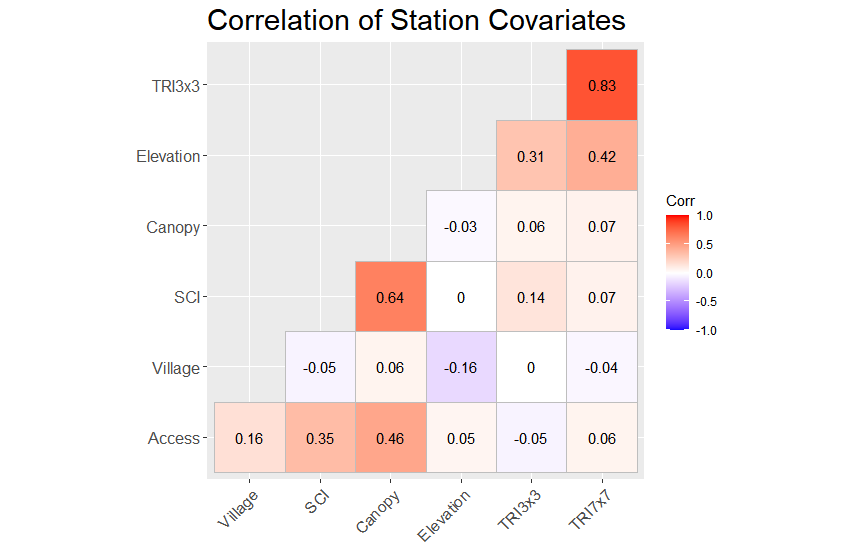


**Table S2**. Summary table of species detected during camera-trap surveys in Pasin and Raplex Forest Management Units and adjacent licensed planted forests, Borneo, including number of detections in each forest landuse type, number of stations in each forest landuse type where a given species was detected, threat status based on *The IUCN Red List of Threatened Species*, and endemism.

|  |  | **All Stations** | | **Natural Forest Management** | | **Licensed Planted Forest** | |  |  |
| --- | --- | --- | --- | --- | --- | --- | --- | --- | --- |
| **Species** | **Scientific Name** | **# detections** | **# Stations detected** | **# detections** | **# Stations detected** | **# detections** | **# Stations detected** | **IUCN status** | **Endemic to Borneo** |
| Bearded pig | *Sus barbatus* | 1105 | 130 | 674 | 90 | 431 | 40 | Vulnerable |  |
| Sambar | *Rusa unicolor* | 640 | 99 | 408 | 68 | 232 | 31 | Vulnerable |  |
| Muntjac^1^ | *Muntiacus sp.* | 1280 | 122 | 950 | 87 | 330 | 35 | *M. atherodes* - Near Threatened *M. muntjak* - Least Concern | *M. atherodes* |
| Mousedeer^2^ | *Tragulus sp.* | 418 | 57 | 269 | 41 | 149 | 16 | Least Concern |  |
| White-fronted langur | *Presbytis frontata* | 11 | 8 | 6 | 4 | 5 | 4 | Vulnerable | ✓ |
| Maroon langur | *Presbytis rubicunda* | 6 | 4 | 5 | 3 | 1 | 1 | Least Concern |  |
| Southern pig-tailed macaque | *Macaca nemestrina* | 1381 | 119 | 881 | 78 | 500 | 41 | Endangered |  |
| Long-tailed macaque | *Macaca fascicularis* | 303 | 59 | 213 | 46 | 90 | 13 | Vulnerable |  |
| Philippine slow loris | *Nycticebus menagensis* | 1 | 1 | 1 | 1 |  |  | Vulnerable |  |
| Sunda clouded leopard | *Neofelis diardi* | 50 | 27 | 34 | 20 | 16 | 7 | Vulnerable |  |
| Bay cat | *Catopuma badia* | 8 | 8 | 6 | 6 | 2 | 2 | Endangered | ✓ |
| Marbled cat | *Pardofelis marmorata* | 16 | 11 | 12 | 8 | 4 | 3 | Near Threatened |  |
| Sunda leopard cat | *Prionailurus javanensis* | 343 | 67 | 298 | 48 | 45 | 19 | Least Concern |  |
| Asian small-clawed otter | *Aonyx cinereus* | 12 | 5 | 10 | 3 | 2 | 2 | Vulnerable |  |
| Yellow-throated marten | *Martes flavigula* | 101 | 50 | 77 | 35 | 24 | 15 | Least Concern |  |
| Malay civet | *Viverra tangalunga* | 1039 | 121 | 754 | 85 | 285 | 36 | Least Concern |  |
| Banded civet | *Hemigalus derbyanus* | 397 | 62 | 306 | 45 | 91 | 17 | Near Threatened |  |
| Hose's civet | *Diplogale hosei* | 9 | 3 | 9 | 3 | - | - | Vulnerable | ✓ |
| Otter civet | *Cynogale bennettii* | 1 | 1 | 1 | 1 | - | - | Endangered |  |
| Masked palm civet | *Paguma larvata* | 56 | 36 | 33 | 25 | 23 | 11 | Least Concern |  |
| Common palm civet | *Paradoxurus hermaphroditus* | 42 | 17 | 37 | 14 | 5 | 3 | Least Concern |  |
| Small-toothed palm civet | *Arctogalidia trivirgata* | 6 | 5 | 5 | 4 | 1 | 1 | Least Concern |  |
| Binturong | *Arctictis binturong* | 11 | 11 | 9 | 9 | 2 | 2 | Vulnerable |  |
| Mongoose^1^ | *Herpestes sp.* | 181 | 71 | 145 | 50 | 36 | 21 | Near Threatened |  |
| Sun bear | *Helarctos malayanus* | 146 | 55 | 92 | 37 | 54 | 18 | Vulnerable |  |
| Banded linsang | *Prionodon linsang* | 4 | 3 | 4 | 3 | - | - | Least Concern |  |
| Sunda pangolin | *Manis javanica* | 46 | 33 | 26 | 21 | 20 | 12 | Critically Endangered |  |
| Tufted ground squirrel | *Rheithrosciurus macrotis* | 29 | 22 | 24 | 17 | 5 | 5 | Vulnerable | ✓ |
| Squirrels^3^ | *-* | 92 | 32 | 71 | 26 | 21 | 6 | - |  |
| Flying squirrels^3^ | *-* | 3 | 2 | 1 | 1 | 2 | 1 | - |  |
| Rats^3^ | *-* | 490 | 61 | 382 | 43 | 108 | 18 | - |  |
| Long-tailed porcupine | *Trichys fasciculata* | 146 | 43 | 131 | 37 | 15 | 6 | Least Concern |  |
| Malayan porcupine | *Hystrix brachyura* | 1001 | 103 | 757 | 70 | 244 | 33 | Least Concern |  |
| Thick-spined porcupine | *Hystrix crassispinis* | 169 | 34 | 144 | 29 | 25 | 5 | Least Concern | ✓ |
| Moonrat | *Echinosorex gymnura* | 1 | 1 | 1 | 1 | - | - | Least Concern |  |
| Tree shrews^3^ | *-* | 234 | 45 | 155 | 33 | 79 | 12 | - |  |
| Bulwer's pheasant | *Lophura bulweri* | 3 | 2 | 1 | 1 | 2 | 1 | Vulnerable | ✓ |
| Great argus | *Argusianus argus* | 319 | 39 | 121 | 30 | 198 | 9 | Near Threatened |  |
| Bornean crested fireback | *Lophura ignita* | 15 | 6 | 9 | 4 | 6 | 2 | Near Threatened | ✓ |
| Crested partridge | *Rollulus rouloul* | 34 | 19 | 25 | 14 | 9 | 5 | Near Threatened |  |
| Bornean ground-cuckoo | *Carpococcyx radiceus* | 1 | 1 | - | - | 1 | 1 | Near Threatened | ✓ |
| Common water monitor | *Varanus salvator* | 8 | 5 | 8 | 5 | - | - | Least Concern |  |
| Rough-necked monitor | *Varanus rudicollis* | 11 | 10 | 7 | 6 | 4 | 4 | Not yet assessed |  |
| Asian giant tortoise | *Manouria emys* | 1 | 1 | 1 | 1 | - | - | Critically Endangered |  |

^1^ Two muntjac species (Southern red muntjac *Muntiacus muntjak* and Bornean yellow muntjac *Muntiacus atherodes*) and two mongoose species (Collared mongoose *Herpestes semitorquatus* and Short-tailed mongoose *Herpestes brachyurus*) were detected during the survey but were grouped together by genus during the identification process.

^2^ We are currently uncertain if both Lesser mousedeer *Tragulus kanchil* and Greater mousedeer *Tragulus napu* are present.

^3^ Records identified according to broader taxonomic groups due to difficulty in discerning species in photos.

**Figure S2.** Effect of natural forest management (NFM), relative to licensed planted forest (LPF), on occupancy probability (mean and 95% Bayesian Credible Intervals, BCI) for 25 medium-to-large bodied terrestrial species, estimated using a community occupancy model fit to camera-trap data from two forest management areas in Sarawak, Malaysian Borneo. Red dots/bars indicate strong association between landuse and occupancy. Effect size was calculated by subtracting the occupancy intercept in LPF from the occupancy intercept in NFM.


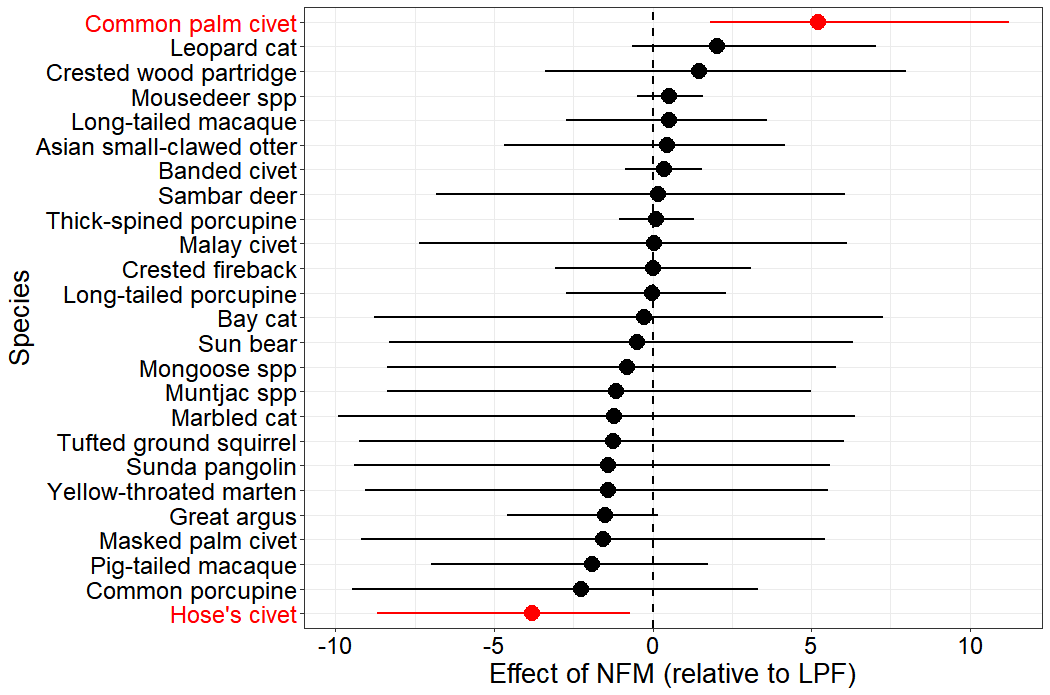


**Figurer S3:** Percentage of area occupied (PAO) between areas of natural forest management (Natural) and licensed planted *Acacia* forest (Planted) for 25 medium-to-large bodied terrestrial species, predicted with a community occupancy model fit to camera-trap data from two forest management units in Sarawak, Malaysian Borneo.


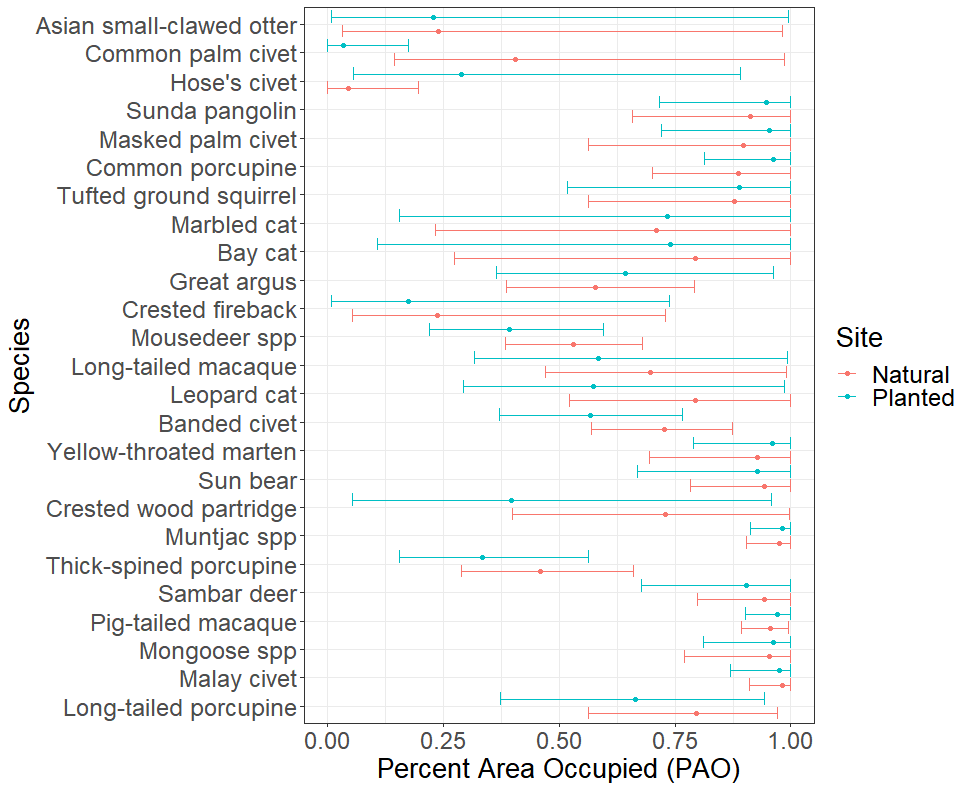

Supplement: Supplementary file 1 — Appendix S1 [file ECE3-12-e9337-s001.docx]
